# Design of a Collaborative Knowledge Framework for Personalised Attention Deficit Hyperactivity Disorder (ADHD) Treatments

**DOI:** 10.3390/children10081288

**Published:** 2023-07-26

**Authors:** Pornsiri Chatpreecha, Sasiporn Usanavasin

**Affiliations:** School of Information, Computer and Communication Technology, Sirindhorn International Institute of Technology, Thammasat University, Pathum Thani 12000, Thailand; pornsirichat@pim.ac.th

**Keywords:** attention deficit hyperactivity disorder, machine learning, knowledge framework, screening tool

## Abstract

Attention deficit hyperactivity disorder (ADHD) is a neurodevelopmental disorder. From the data collected by the Ministry of Public Health, Thailand, it has been reported that more than one million Thai youths (6–12 years) have been diagnosed with ADHD (2012–2018) This disorder is more likely to occur in males (12%) than females (4.2%). If ADHD goes untreated, there might be problems for individuals in the long run. This research aims to design a collaborative knowledge framework for personalised ADHD treatment recommendations. The first objective is to design a framework and develop a screening tool for doctors, parents, and teachers for observing and recording behavioural symptoms in ADHD children. This screening tool is a combination of doctor-verified criteria and the ADHD standardised screening tool (Vanderbilt). The second objective is to introduce practical algorithms for classifying ADHD types and recommending appropriate individual behavioural therapies and activities. We applied and compared four well-known machine-learning methods for classifying ADHD types. The four algorithms include Decision Tree, Naïve Bayes, neural network, and k-nearest neighbour. Based on this experiment, the Decision Tree algorithm yielded the highest average accuracy, which was 99.60%, with F1 scores equal to or greater than 97% for classifying each type of ADHD.

## 1. Introduction

Attention deficit hyperactivity disorder (ADHD) is a complex mental health disorder that affects children aged six to twelve years [1,2,3]. ADHD is classified into three main subtypes: inattentive, impulsive–hyperactive, and a combination of the two. An individual with the inattentive subtype has difficulty paying attention and staying focused, while an individual with the impulsive–hyperactive type not only often feels restless and yields to impulses easily compared to other children in the equivalent development stage, but also has difficulty paying attention, similar to the inattentive subtype [1,2,4,5]. To diagnose a pupil with any of the subtypes mentioned earlier, they must show at least six of the nine given symptoms for at least six months [2,6,7].

ADHD is a severe neurodevelopmental disorder that has multiple aetiologies, but it is quite complicated to pinpoint a single factor that might be the cause. Mainly, it is caused by a combination of environmental and genetic factors. Environmental factors include lead poisoning from maternal smoking during pregnancy, a less supportive home environment, and recent studies even show that the number of hours of watching television or using other media devices might contribute to environmental factors causing ADHD [4,5,8,9]. As for the genetic factors, these may be due to parental genes, which influence or regulate the production of neurotransmitters in a child’s brain during their development. The lack of neurotransmitters like dopamine (impulsiveness) or norepinephrine (attention) might result in the child being diagnosed with ADHD [3,4,10,11,12,13,14]. Unfortunately, at present, there is no available cure for ADHD, but if diagnosed early, help is provided as soon as symptoms surface, risks are detected, or ADHD predisposition is diagnosed. EI aims to help, to the fullest potential, promote physical, emotional, mental, social, and intellectual development. If a child is being supported from the beginning, the child is deemed to have the development of an average individual. In addition, the action reduces the cost and burden incurred [2,7,15,16].

If behavioural syndrome goes untreated, there might be problems for individuals in the long run. The patients might be at risk of other health conditions. They would also suffer from decreased social life quality, unsatisfactory academic performance, unpleasant relationships, comorbid psychiatric conditions, and much more. If not treated until adulthood, the symptoms may affect the health and lifestyle of the patients in many aspects. According to research about the persistence rate of ADHD into adulthood, the psychiatric comorbidities of adult ADHD, and the risk of serious adverse outcomes, such as criminality and mortality, ADHD should no longer be viewed only as a disorder primarily affecting the behaviour and learning of children [17,18]. ADHD should also be regarded as a significant health condition that confers an increased risk for early death due to suicide. In addition, although nearly one-third of children with ADHD will continue to fulfil norm-referenced criteria for ADHD as adults, most will also have at least one mental health problem in adulthood.

Children with ADHD experience far more obstacles compared to their average counterparts. While many children diagnosed with ADHD receive some special school services to improve their learning environment and experience, this might not always be the case [19]. Consecutively, parents must observe their children’s behaviour when at home, and teachers can help by monitoring and evaluating the behaviour while individuals are at school. For the assessment, teachers and parents can use the questionnaires of the DSM-5 standard [4,20], particularly the Vanderbilt ADHD Diagnostic Rating Scale (VADRS) (Thai version) [21]. Furthermore, teachers should become involved, as they know the students personally and academically. In addition, there are chances that the child’s ADHD symptoms could be neglected and considered as habits by their parents. Therefore, having multiple perspectives and integrating all the results from different informants would be most optimal. With the expertise and evaluation of the assessment tests, physicians could then recommend and guide teachers and parents with methodologies and strategies which would ensure that, if followed, the children would be able to manage their symptoms [19,21,22].

The primary purpose of this research was to design a collaborative knowledge framework for personalised ADHD treatments. The main objectives included (1) a design of a collaborative framework and a screening tool for ADHD symptoms that could be used by medical professionals, parents, and teachers, and (2) the introduction of a practical algorithm for the classification of the types of ADHD with sets of recommended individual behavioural therapies and activities for different types of ADHD. To find an appropriate classification technique for the types of behavioural syndrome and address the need for such a machine learning algorithm in Thailand, we use machine learning to help with early intervention and learn student behaviour to assess the risk of having ADHD and find a solution; this will help children and those around them to heal and increase their chances of recovering. With the advancement of machine learning, we can classify symptoms more precisely and support large amounts of data, so there are advantages to using machine learning over queries alone.

We applied different machine learning algorithms, compared the results, and utilised the best algorithm in our framework. The expected outcome of our proposed framework was to provide an effective way to classify the types of behavioural syndrome and to recommend appropriate treatments and therapies based on the individual’s behaviour.

For example, the prevalence of ADHD worldwide ranges between 0.1 and 8.1%. In Thailand, the prevalence is between 4.2 and 8.1% [23,24]. The prevalence of ADHD in children in Africa is 7.47% [25]. In 2016, an estimated 6.1 million U.S. children 2–17 years of age (9.4%) had received an ADHD diagnosis and 5.4 million children currently have ADHD [26]. The difference is due to the variety of population groups, ADHD study tools, and research procedures. For Thailand, only regional-level research was conducted.

This paper is organised as follows: Section 2 summarises previous studies that led to the motivation of this research. Section 3 describes the proposed approach and methods utilised in this research. Section 4 explains our experimental results, and Section 5 provides a discussion and conclusion for this work.

## 2. Related Work

Children are taken to the doctor for the early diagnosis and treatment of ADHD when behaviour resembling ADHD symptoms is observed during the evaluation. For instance, a child is much more mischievous than usual and exhibits emotional outbursts and lacks learning concentration. Hence, ADHD should be diagnosed based on an evaluation of learning, behavioural, and emotional issues. When determining the diagnosis of ADHD, it is critical to consider the following evaluations: (1) an evaluation of the patient’s history and current conditions, (2) an evaluation by the parents and teachers, (3) a psychological assessment, and (4) discussion and treatment recommendations [1,2,5,15,20,27].

The development of technology related to health informatics is underway. This includes a decision support system (DSS) and patient follow-up care in the healthcare system [28]. Effective systems require cohesive and synergistic thinking, including collaborations between doctors and patients for an appropriate design [29]. Thus, it is important to have a DSS for the ADHD screening and diagnosing process for young patients with ADHD. De Silva et al. [30] proposed the development of a DSS for ADHD based on knowledge of the patterns from past screening support systems. This was able to distinguish children with ADHD from other similar children’s behavioural disorders. In addition, in designing a system, there would be important aspects that would need to be considered, such as diagnostic and monitoring approaches.

The Vanderbilt ADHD Diagnostic Rating Scale (VADRS) is a well-known standardised screening approach that aids doctors in making ADHD diagnoses based on the Diagnostic and Statistical Manual of Mental Disorders, Fifth Edition (DSM-5) standard [1,2,15,19] and assessing comorbid conditions. VADRS includes 18 symptoms described in the DSM-5. VADRS separates the teachers’ (VADTRS) and parents’ (VADPRS) versions of the assessment forms [22,31,32,33,34]. Additionally, VADRS contains comprehensive information to make an appropriate DSM-5-based diagnosis of ADHD and screens for common commodities. Moreover, VADRS has scales that allow for measuring the comorbidities by externalising and aiding in providing appropriate treatment plans. The only setback is its lack of data validity, data supporting stability, and discriminant validity in evaluation and treatment [22,33,34,35].

Contemporary machine learning techniques are used in several healthcare applications [28,36,37,38]. They are employed to predict future diseases and offer a desirable decision from a data set. We describe and compare the advantages and disadvantages of machine learning in Table 1.

Many researchers have used machine learning algorithms to indicate diseases such as liver disease (logistic regression with 95.8% accuracy) [38], breast cancer (support vector machine with 99% accuracy) [39], and Alzheimer’s disease (neural networks with 98.3% accuracy) [40]. There are previous studies that have also proposed various machine learning algorithms to predict and classify ADHD. Krishnaveni and Radhamani [41] used Naïve Bayes and the J48 classifier as machine learning techniques with questionnaires as a tool to classify ADHD. The results achieved a classification accuracy of 100%. Additionally, Deping Kuang and Lianghua He [42] utilised the deep belief network (DBN) with a magnetic resonance imaging (MRI) method to indicate ADHD, which achieved a classification accuracy of 85%. Likewise, Öztoprak et al. [43] used the Disruptive Behaviour Disorders Rating Scale Form (DBDRS), for a study that employed a Decision Tree (DT) (CART), DT (CHAID), and neural network to yield prediction accuracies of 69.1%, 70.6%, and 61.8%, respectively. Bo Miao and Yulin Zhang Das [44] used the feature selection algorithm of three methods, for which accuracies of 77.92%, 80.52%, and 98.04% were obtained for the relief algorithm (Relief), verification accuracy (VA-Relief), and minimum redundancy maximum relevance (mRMR), respectively. Likewise, Khanna and Das [45] used the feature selection algorithm and achieved a prediction accuracy of 82.10%. Jian Peng et al. [46] used a neural network algorithm with MRI to indicate the types of ADHD, yielding 72.89% accuracy. Cordova et al. [47] incorporated data from the DSM-IV-TR and used the Random Forest algorithm to predict ADHD and the types of autism spectrum disorder (ASD) with an accuracy of 72.70%. Radhamani and Krishnaveni [48] employed a hybrid approach integrating a support vector machine (SVM) and DT algorithms. The hybrid model gave an accuracy of 100% for classification and prediction. Parashar et al. [49] used the SVM, Random Forest, and AdaBoost Classifier (applied algorithm) to predict the types of ADHD, and they obtained an accuracy of 58%, 82%, and 84%, respectively. Furthermore, Lizhen Shao et al. [50] used the SVM and MRI and obtained an accuracy of 92.68%.

In the case of this research, the aim was to examine and apply the most suitable algorithm to predict and classify the types of ADHD by using the collected and observed data from teachers and parents in real cases. The method for the data collection is also explained in this paper. Compared to the previous work, different assessment techniques were used for screening and predicting the types of ADHD and oppositional defiant disorder (ODD). Input data were obtained by using a standardised screening tool based on the behaviour and culture of Thai children (VADRS), which was evaluated by a group of teachers. In this work, the classification results and the physicians’ diagnoses were compared to validate our results. Based on the previous study, the four techniques (DT, Naïve Bay, neural network, and k-nearest neighbour (KNN)) that provided highly accurate results were examined and tested with our data set. The classification results from these four models were compared, and we selected the model that could return the most accurate result to be used in our framework. The DT is known to support non-linear data, and it could provide highly accurate results with a trained model. The results from the DT were straightforward, and the model could be improved easily based on the interpretation of the results. The Naïve Bay algorithm is a data mining classifier. It offers extensive features and data that could also be used to classify data that had multiclass characteristics. The neural network algorithm is flexible and simple to use with a few parameters to adjust. It can simulate problems and remember a series of input–output pairs that are complex and cannot be replicated in a probabilistic way. The KNN is also a straightforward technique that can be used for data classification.

In this work, we applied the mentioned four models to the input data that we had collected by using the developed screening tool in our framework. This input data were provided by a group of teachers who regularly observed the behaviour of their students in class. The input form was developed based on the VADRS. The results of each model were validated and compared with the results of the diagnoses from the doctors. Thus, we applied the model with the best results for the classification module of the types of ADHD in our framework.

## 3. Research Methodology

**Figure 1:**
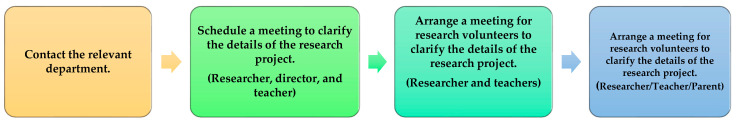
Participant recruitment process.

This section presents the proposed research methodology, research framework, and classification technique for the types of ADHD, and the recommendation system based on behavioural therapy and activities for ADHD children. Before starting the process of the research methodology, we approved the ethics via the Human Research Ethics Committee of Thammasat University (Science) (HREC-TUSc)) and the participant recruitment process in Figure 1.

Figure 1 describes the selection and recruitment process of volunteers. The steps are as follows:

1. Ban Rat Niyom School (Jor Prayoon Upatham) was contacted with detailed documents about research work. The documents related to research work were presented to the school’s director.
2. After receiving approval from the school’s director, the researcher arranged a meeting to explain the details of the research process, activities, recruitment, and other information related to conducting research for the upcoming project.
3. Volunteer recruitment for teachers was conducted. After that, an appointment was made to meet and clarify the research implementation requirements. Documents relevant to the study and the activities to take place were presented throughout the research project.
4. The supervised teachers chose students. Then, they sent the parents the participant data sheet and consent letter. If the parents had any doubts regarding student participation, teachers could contact researchers to arrange meetings for clarification.

Participants were selected according to the following inclusion criteria.

(Inclusive criteria for teachers):
  - Only homeroom teachers were selected, and they must have the following qualifications.
  - The teachers must teach and supervise children of age 6–12 years old who study at the primary level (grade 1–6) at Ban Ratniyom School (Jorprayoon Upatham).
  - The teachers have knowledge of and understand information about ADHD in children. They can assess and observe student behaviour in their supervising classes and are able to use a tool to screen behavioural/emotional problems, including the Strengths and Weaknesses Scale (SDQ, Teacher Student Behaviour Assessment Scale).
(Inclusive criteria for students):
  - Students must be 6–12 years old and study at the primary level (grade 1–6) at Ban Rat Niyom School (Jor Prayun Upatham). They are in the class of the teachers under the criteria stated above. The participating teachers selected students for this study.
(Inclusive criteria for parents):
  - Parents of the selected students, who were willing to participate, were included.

The exclusion criteria for research volunteers are as follows:

- Teachers who cannot participate in activities during the specified period of the research project were excluded.
- Teachers who could not assess and observe students’ behaviours in their supervised classes according to the specified criteria and within the duration of the research project were excluded.
- There were no exclusion criteria for students and parents.

### 3.1. Design and Development of the Proposed Collaborative Framework

In this framework, there were three types of participants: teachers, parents, and doctors. The framework provided a collaborative tool for all participants to provide collaborative information based on the VADRS for preliminary assessment.

#### Workflow of the Collaborative Framework

The roles and responsibilities of the participants in the framework are shown in Figure 1. In this framework, the teacher evaluated the students using the VADRS, and the parents assessed their children using the same screening scale. The doctor determined and validated the results. The doctor could request to have a discussion and consultation with the teacher or parent for the appropriate treatment. The system used recorded information from the teachers and parents to perform the classification of the types of ADHD and provided recommendations for behavioural therapy for each student based on his/her type of ADHD. The treatments in the recommendation system were pre-input according to the medical recommendations based on the different types of ADHD (Figure 1).

**Figure 2:**
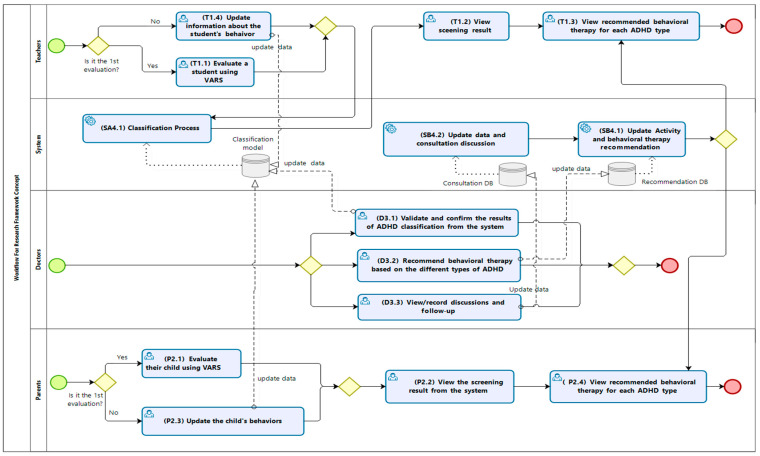
Workflow of the proposed framework.

Figure 2 shows the workflow for this research framework. The solid arrows indicate the sequence and direction of the process. The dashed line represents the data flow between the process and the database in the system. The overall process is described as follows:

- The role of the teacher was to evaluate students using the VADRS (refer to T1.1 in the framework) and view the screening of the result (T1.2). The teacher could also view the recommended behavioural therapy for each type of ADHD from the system (T1.3). The teacher could consult with the doctor if he/she had any questions about how to apply the therapy or activity based on the recommendations from the system. After the teacher applied some therapies and/or treatment activities to the student, the teacher could update the information about the student’s behaviour in the system for further assessment (T1.4). The updated data would also be used for updating the classification model for improvement.
- The parents could evaluate their child using the same VADRS (P2.1) and view the screening result from the system (P2.2). The parents could update the child’s behaviour in the system for further assessment (P2.3) and view the recommended behavioural therapy for each type of ADHD from the system (P2.4).
- The doctors viewed and confirmed the results of the classification of ADHD that was returned by the system (D3.1) and recommended behavioural therapies based on the different types of ADHD (D3.2). They could view and record the discussions (D3.3) and give the teachers consultation from the system (D3.4) with follow-ups (D3.5).
- Our system consisted of three processes comprising the classification process (SA4.1), the update process for data and consultation discussion (SB4.1), and the update process for the activities and recommendations for behavioural therapy (SB4.2).

This section provides a detailed explanation of SA4.1, SB4.1, and SB4.2 in Figure 2, respectively.

### 3.2. The Classification Process (SA4.1)

The classification was proposed based on the use of the VADRS and machine learning techniques. The results from this classification process were compared with the results evaluated by doctors who were consultants in child and adolescent psychiatry and developmental behavioural paediatrics. The following processes were performed to implement the classification process used in this framework:

(a) Data collection and analysis process

The main data set used in this work was collected from the data provided by the teachers and parents. The system automatically generated a set of questionnaires based on the VADRS and allowed the teachers and parents to evaluate their children via the system. After the system received the data from the teachers and parents, it generated the data set files that were used for the classification process. In this work, we obtained data for 420 cases.

(b) Model generation process

After the data set files were created from the previous step, these data set files were pre-processed to remove any duplicates, missing data, and inconsistencies. We exploited 52 attributes of the VADRS for learning and generating a model. To do so, the data set was imported and divided into two parts with a ratio of 80% for training the model and 20% for testing the model. The development of the model by training the data process utilised the feature extraction approach. The SelectPercentile module from the sci-kit-learn tool decreased the number of attributes and selected only the important features (selection) or converted features (transformation) to reduce the dimensions. After the data were declined dimensionally, they were processed for classification. The performance of the model was tested with the data set by specifying the percentage of the properties to be chosen rather than the number of properties to be determined. The top N% percentile was chosen to acquire the procedure of the whole 10 properties. This could be summarised as follows:

1. Set SelectPercentile = N%.
2. No. of the remaining attributes ≤ N%.
3. Update the set of attributes based on the SelectPercentile.
4. Return the attribute selection result.

Based on the above procedure, we could reduce the matrix dimension by using the SelectPercentile of 40% for optimisation. A low percentage led to fewer attributes that were utilised to create the model, thus making it unable to extract the data accurately. Still, a high percentage resulted in the model structure having a high complexity. The value of 40 gave the best result from the experiments, with eight remaining attributes being significant for the results of the experiment. Next, we applied various machine learning algorithms and tested each algorithm separately to determine the performance of each model. These algorithms were the DT, Naïve Bayes, neural network, and KNN.

(c) Prediction process

This sub-process was to query the model from the database system. After selecting the model algorithm, the system prediction was saved to the database, and the predicted model algorithm was yielded.

(d) Verification of the result of the predicted model

To verify the results from our classification models, the models’ results were reviewed and validated by the doctors. These doctors specialised in child and adolescent psychiatry and developmental behavioural paediatrics. By comparing the accuracy and performance of all the models, the most appropriate classifier was discovered and selected for classifying the types of ADHD in our framework.

### 3.3. The Activity and Behavioural Therapy Recommendation Process (SB4.1)

This section explains how the system recommended activities and behavioural therapy for ADHD children based on the different types of ADHD. In this process, the system recommended the appropriate activity and behavioural therapy based on the classified type of ADHD the child shows. The information is summarised as follows [13,51,52,53].

Table 2 shows recommending activities for ADHD children by type as defined below (Recommend by the doctor).

1. Mix-type is a symptom of the hyperactivity–impulsivity and inattention type of ADHD. The activities for this type focus on organisation and discipline activities (AOCD) and medication activities (AMOD).
2. Hyperactivity is a symptom of a hyperactivity–impulsivity type of ADHD. The activities for this type include organisation and discipline activities (AOCD).
3. Inattention is a symptom of a lack of concentration. The activities for the children involve increasing concentration (AIC).
4. ODD is oppositional defiant disorder ADHD. The activities focus on those that can control behaviour (ACB).

To implement this process, the following sub-processes were performed.

(a) Data collection and algorithm design

In this sub-process, three tasks were performed.

(i) Questionnaires, interviews, focus group discussions, and social media were employed to collect the data sources from the parents, teachers, and doctors. This information was determined and used for designing the recommendation process and the algorithm used in the framework.
(ii) We studied and evaluated the classification algorithms for the classification of the types of ADHD based on the VADRS [4,20].
(iii) We designed an algorithm to provide the appropriate recommendations for behavioural therapy and treatment activities based on the different types of ADHD. The verified information on the behavioural therapy and treatment activities was provided by the doctors, and it was pre-input into the system for the recommendation process.

(b) Review the recommended information and algorithm

The recommended information and algorithm were reviewed and validated by the doctors.

- Update data and consultation discussion (SB4.2)

The teachers and parents could have consultations with the doctors for more detailed recommendations and discussions about the recommended therapies and activities. After the discussions, which were conducted manually, the doctors updated the information in the system, so the teachers and parents could view the doctor’s recommendation and discussion details via the system.

## 4. Experiments and Results

### 4.1. Analysis of the Classification Results

The classification results and the results from the behavioural therapy-based recommendation system for ADHD children from the experiments are presented in this section.

To evaluate the classification models, a confusion matrix was widely used for the performance measurement. The confusion matrix was a table of size *n* by *n* that was given for the *n* classes. If the incident was positive and classified as such, it was considered a true positive (TP). It was considered a false negative (FN) if it was labelled as negative. If the incident was negative and characterised as such, it was considered a true negative (TN). If it was classed as positive, it was considered a false positive (FP) [50,51,52,53,54].

True positive and true negative are performance matrices for classification performance for machine learning models. In a classification problem with five classes, the concepts of true positive and true negative can be a bit different compared to binary classification. TP represents the true positives, indicating the instances correctly classified for each class. FN (false negative) represents the instances that belong to a particular class but are incorrectly classified as one of the other four classes. Each cell in the matrix that is not a TP represents an FN for that specific class. There are no TN (true negative) values explicitly defined in this case since they are related to correctly classifying instances as not belonging to the specific class in consideration. Based on the experimental results, we chose a classification model that provided the best performance and used this model for our screening tool to classify ADHD types. After the system identifies the ADHD type, the system recommends appropriate treatments and activities based on the ADHD type. 

For a confusion matrix for a two-class classification problem, the numbers along the diagonal, from upper-left to lower-right, reflect the correct decisions, whereas the numbers outside of this diagonal represent errors. The TP and TN values estimate a classifier’s overall accuracy. Other aggregated performance indicators were calculated using recall (sensitivity), specificity, and the F-measure. As defined below, the performance measurements were calculated.

(1) $$\mathrm{Classifier}\;\mathrm{Accuracy}=\frac{\mathrm{TP}+\mathrm{TN}}{\mathrm{TP}+\mathrm{TN}+\mathrm{FP}+\mathrm{FN}}$$

(2) $$\mathrm{True}\;\mathrm{Positive}\;\mathrm{Rate}\;(\mathrm{TPR})=\frac{\mathrm{TP}}{\mathrm{TP}+\mathrm{FN}}$$

(3) $$\mathrm{True}\;\mathrm{Negative}\;\mathrm{Rate}\;(\mathrm{TNR})=\frac{\mathrm{TN}}{\mathrm{TN}+\mathrm{FP}}$$

(4) $$\mathrm{Recall}\;(\mathrm{RC})=\frac{\mathrm{TP}}{\mathrm{TP}+\mathrm{FN}}$$

(5) $$\mathrm{Precision}\;(\mathrm{PR})=\frac{\mathrm{TP}}{\mathrm{TP}+\mathrm{FP}}$$

(6) $$F1-\mathrm{score}\;(F1)=\frac{2*\left(\mathrm{Precision}+\mathrm{Recall}\right)}{\left(\mathrm{Precision}+\mathrm{Recall}\right)}$$

(7) $$\mathrm{Average}\;\mathrm{Accuracy}=\frac{\sum_{i=1}^{l}\frac{{\mathrm{TP}}_{i+}{\mathrm{TN}}_{i}}{{\mathrm{TP}}_{i}+{\mathrm{FN}}_{i}+{\mathrm{FP}}_{i}+{\mathrm{TN}}_{i}}}{l}$$

The classifier accuracy (Equation (1)) is a measurement used to assess which model would be the most appropriate at recognising the correlations and patterns between the variables in the data set based on the inputs (or training data). A good classification model should have high accuracy. Equation (2) shows the TPR or sensitivity, which refers to the probability of a positive test, conditioned on truly being positive.

Equation (3) shows the TNR or specificity, which refers to the probability of a negative test, conditioned on truly being negative. Contrary to the other equations, a more favourable result for this equation was closer to 0. A result of 0 referred to a 0% chance of a model predicting a case incorrectly.

Equation (4) shows recall (RC). It is also known as sensitivity or TPR, which is the measure of our model correctly identifying the TPs.

Equation (5) shows precision (PR), which is the ratio between the TPs and all the positives.

Equation (6) shows the F1 score (F1), which is a metric that takes into account both precision and recall precision.

Equation (7) shows the average accuracy, which is the average effectiveness per class of the classifier.

Table 3 shows the summary of the cross-validation of the four classifiers. It also compares the outcomes between the system results and the validated results from the doctor. For this experiment, there were 336 records of training data (80%), 84 records of test data (20%), and 420 cases of doctor-confirmed outcomes.

In Table 4, the results of the four classifiers were tabulated for comparison. Based on the results (Table 5), the DT and neural network models provided the highest accuracy. The DT method and neural network algorithm provided an accuracy of 99.57%, while the KNN algorithm achieved up to 98.72%. The Naïve Bay algorithm yielded an accuracy of 94.47%.

The average accuracy of the classification was 99.60% for the DT and neural network models. The KNN algorithm provided an average accuracy of 98.40%, whereas the Naïve Bayes yielded 94.00%. Furthermore, as shown in Table 3, the DT and neural network models produced the same values of the TPR, TNR, PR, RC, AC, and F1 for all types of ADHD. Both models had values greater than 97% for the TPR, RC, and F1. The PR of 95% and the TNR of 2% indicated the low probability of TN testing. 

The classification results of the KNN algorithm showed 100% TPR for all cases except for 94% for the mix-type. A TNR of 0% was also yielded for cases except for inattention, for which the model could predict the case incorrectly with a 5% chance. For PR, only inattention yielded results of 86%, while it had a 100% ratio for the TPs and all the positives for the other types of ADHD. RC showed 100% for all cases, except for 94% for the mix-type. For AC and F1, only the mix-type and inattention did not have a result of 100%. 

### 4.2. Analysis of the Recommendation Process

This section explains how the system recommends activities and behavioural therapy for ADHD children based on the different types of ADHD. In this process, we used the DT algorithm for finding the appropriate recommendations for each type of ADHD. 

From our experiments, the average classification accuracy offered by the Decision Tree and neural network models is equivalent to and better than other models. However, the main reason that we chose Decision Tree is because we found that it also provides better computation time compared to the neural network model. Table 6 shows an example of computation time offered by the Decision Tree and neural network from our experiments. 

#### The Decision Tree Graph

As the average accuracy of the classification was 99.60% for the DT and the neural network models, the DT algorithm was selected because the value of the F1 was greater than 97%, while PR was 95%. Its FPR of 0.02 indicated a low probability of a wrong prediction. From several experiments of the data sets, we found that the DT algorithm gave predictive accuracy close to the results of each experiment (Table 7).

**Figure 3:**
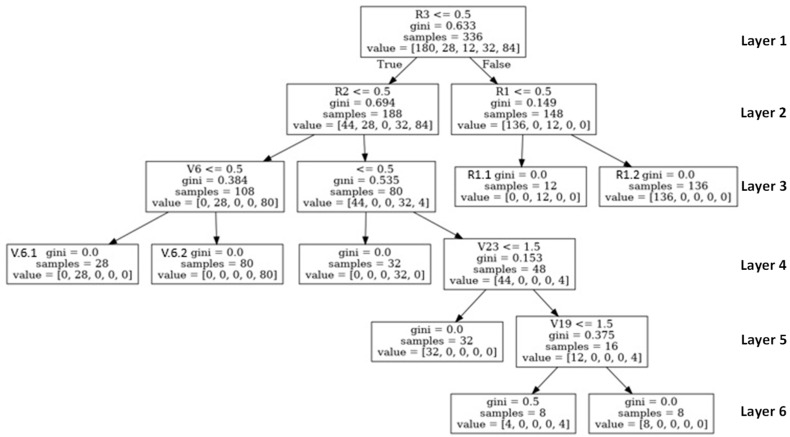
The Decision Tree graph.

The description for the parameter in the tree graph (Figure 3) is as follows:

(1). The samples parameter is the number of data items compatible with that node, so as the decision moved down the depth of the tree, the number of samples of a node in each layer tended to decrease over time.
(2). Gini indicates the “purity” of a node. Where Gini = 0, this infers that all the data items in the node belong to the same class. In comparison, Gini = 0.5 indicates that the data items in the node belong to two similar types, which represent the values, such as the value of R1 = [136, 0, 12 0, 0] in the child node to the right of the root node. This infers the 148 entries of 15 at this node condition. If the answer was false (child node left R1) and the value was [0, 0, 12, 0, 0], there would be 12 entries in the ODD classification. However, if the answer was true and the value was [136, 0, 0, 0, 0] (child node right R1), there would be 136 entries in the mix-type classification. This assumes that the data meeting this node’s condition is in the ODD and mix-type classifications.
(3). Value is used to indicate the class of the predicted activities by the types of ADHD. The activities of the five classes were mix-type (index [0]), non-ADHD (index [1]), ODD (index [2]), hyperactivity (index [3]), and inattention (index [4]).

We tested the DT algorithm for processing the recommendations of the activities for ADHD children. The results are shown in Table 5. The PR and TNR were 0.95 and 0.02, respectively, and the average accuracy was 0.996. This result was reviewed and validated by doctors, and the accuracy was acceptable.

## 5. Discussion and Conclusions

In this research, we aimed to overcome the mentioned problems by proposing a methodology and framework that teachers or parents could use to evaluate and screen their children’s behaviour and determine if they were consistent with the types of ADHD. The framework provided recommendations for the appropriate treatments for different types of ADHD children. The expected outcome of our proposed framework was to provide an effective way to screen and classify the types of ADHD and recommend appropriate treatments and therapy based on individual behaviour.

The average classification accuracy of 99.60% was achieved by the DT and neural network algorithms. On the other hand, the KNN approach had an average classification accuracy of 98.40%, whereas the Naïve Bay technique had an average classification accuracy of 94.00%. Although the DT and neural network algorithms returned similar accuracy, in this work, we chose the DT algorithm as the main model for the classification of the types of ADHD and recommendations of the activities because the tree structure could be easily explained to all the participants for analysis, change, and future improvement. In addition, the performance, when adding a new data set to the training model, required a shorter time compared to the time required to fine-tune the neural network. 

The limitation of our research is data collection. During our data collection process, the COVID-19 pandemic was seriously occurring in Thailand and all schools were closed, so it was very difficult to collect data from all participants in the study. This resulted in the small amount of data we used in this work. Also, due to the pandemic situation, we could not directly interview each student to obtain the child’s opinion, which we believe can be valuable and useful for further analysis. Therefore, in our future work, we plan to improve our research by gathering more data from various groups of children such as children in big cities, children in rural areas, etc. Moreover, we will improve our tools, which would better support communications among participants (teachers, doctors, parents) to share information and follow up cases. In Thailand, the employment of machine learning technology in the children’s health care system is limited. This is especially true for ADHD. Moreover, when a child is found with a condition or a tendency to have ADHD, the child relies on many elements to support the treatment, including access to disease information, medical treatment, specialised doctors related to psychiatry, travelling method to the hospital, and unforeseen expenses incurred by the family.

For future work to improve our framework, we would like to generate and expand the data collection and conduct further experiments to design and build more efficient algorithms of the sub-activities based on different types of ADHD. Although the current work could achieve high accuracy for classifying the types of ADHD, some cases would still need to be improved (e.g., classifying an inattention type and mix-type). We also plan to train the model for different scenarios and try to enhance the model’s accuracy and UX/UI design based on feedback from the users (e.g., teachers and doctors).

## Figures and Tables

**Table 1:** Comparison of advantages and disadvantages of machine learning models.

No	Classification Technique	Description	Pros	Cons
1	Decision Tree	A Decision Tree is a supervised learning technique that can be used for both classification and regression problems, but mostly it is preferred for solving classification problems.	- Structured data/Unstructured data - easy implementation	- slight variation in data can lead to a different decision tree - does not work well with small data
2	Naïve Bay	The KNN algorithm classifies data by comparing information of interest to others. The algorithm returns a result based on the information that is most similar to the information of interest.	- Structured data/Unstructured data - easy implementation - high computation efficiency, classification rate, and accuracy.	- precision of the algorithm decreases with fewer data - an extensive number record is required for accuracy
3	KNN	The Naive Bay algorithm is a data mining classifier. The technique was developed based on the principle of Probably Naïve Bayesian Classification. It is used to analyze the probability of an unprecedented event from occurred events.	- Unstructured data - suitable for multimodal class - If the decision-making conditions are complex, this approach can create efficient models - a small dataset and the data is noise-free and labeled.	- excessive time to find the nearest neighbors in an extensive training data set - performance of the algorithm depends on the number of dimensions used
4	Neural Network	This algorithm is one of the data mining techniques. It is a mathematical model for processing information with a connected computation (Connectionist). The algorithm is used to simulate the functioning of neural networks in the human brain to create a tool capable of learning pattern recognition, knowledge extraction (Knowledge Extraction), and the human brain capabilities.	- Structured data/ Unstructured data - simple to use with a few parameters to adjust - applicable to a wide range of problems in real life	- requires high processing time if the neural network is large - difficult to know the required number of neurons and layers

**Table 2:** Recommended activities and behavioural therapy for different types of ADHD.

No	ADHD Type	Activities	Description	Example for Activities
1	Mix-type	AOCD and AMOD	Organization and Discipline Activities and Medication Activities	Play toys or games that require concentration. Training the child to play with one toy at a time will help the child to concentrate on playing longer and Listen—play good music for concentration.
2	hyperactivity	AOCD	Activities Organization/Discipline Activities	Activities that require calmness, such as building blocks of wood or making towers of coins, Feed the eggs with a spoon.
3	inattention	AMOD	Activities Medication Activities	Activities that use distance, such as throwing a ball into the basket, threading the needle, stringing the beads, or stringing the garland,
4	ODD	ACB	Activities Control Behavioral	Create a daily schedule of activities, such as doing homework before play.
5	Non-ADHD	NO-Activities	Non-ADHD	General activities that increase concentration, such as reading stories.

**Table 3:** Statistical data analysis of the ADHD classes.

No	Type of ADHD	Data	Number of Data	%	All Data
0	Mix -Type	train	188	80	235
		test	47	20	
1	Non-ADHD	train	28	80	35
		test	7	20	
2	ODD	train	12	80	15
		test	3	20	
3	hyperactivity	train	32	80	40
		test	8	20	
4	inattention	train	76	80	95
		test	19	20	

**Table 4:** Comparison of the results of the four classifiers.

No	Type of ADHD	Test	Decision Tree				KNN				Naive Bayes				Neural Network			
			Cor	%	Inc	%	Cor	%	Inc	%	Cor	%	Inc	%	Cor	%	Inc	%
0	Mix-type	47	46	97.87	1	2.13	44	93.62	3	6.38	34	72.34	13	27.66	46	97.87	1.00	2.13
1	Non-ADHD	7	7	100.00	0	0.00	7	100.00	0	0.00	7	100.00	0	0.00	7	100.00	0.00	0.00
2	ODD	3	3	100.00	0	0.00	3	100.00	0	0.00	3	100.00	0	0.00	3	100.00	0.00	0.00
3	hyperactivity	8	8	100.00	0	0.00	8	100.00	0	0.00	8	100.00	0	0.00	8	100.00	0.00	0.00
4	inattention	19	19	100.00	0	0.00	19	100.00	0	0.00	19	100.00	0	0.00	19	100.00	0.00	0.00
Total (case)		84		99.57		0.43		98.72		1.28		94.47		5.53		99.57		0.43
% Total Cases			99.57				98.72				94.47				99.57			

Note: Cor = no. of correct results and Inc = no. of incorrect results.

**Table 5:** Performance comparison of the four classifiers.

No	Type of ADHD	Decision Tree						KNN						Naive Bayes						Neural Network					
		TPR	TNR	PR	RC	AC	F1	TPR	TNR	PR	RC	AC	F1	TPR	TNR	PR	RC	AC	F1	TPR	TNR	PR	RC	AC	F1
0	Mix-type	0.98	0	1	0.98	0.99	0.99	0.94	0	1	0.94	0.96	0.97	0.72	0	1	0.72	0.85	0.84	0.98	0	1	0.98	0.99	0.99
1	Non-ADHD	1	0	1	1	1	1	1	0	1	1	1	1	1	0	1	1	1	1	1	0	1	1	1	1
2	ODD	1	0	1	1	1	1	1	0	1	1	1	1	1	0	1	1	1	1	1	0	1	1	1	1
3	hyperactivity	1	0	1	1	1	1	1	0	1	1	1	1	1	0	1	1	1	1	1	0	1	1	1	1
4	inattention	1	0.02	0.95	1	0.99	0.97	1	0.05	0.86	1	0.96	0.93	1	0.2	0.59	1	0.85	0.75	1	0.02	0.95	1	0.99	0.97
Average Accuracy		0.996						0.984						0.94						0.996					

Note: TPR = rate of true positives; TNR = true negative rate; PR = precision; RC = recall; AC = accuracy; and F1 = F1 score.

**Table 6:** Comparison of model time between the Decision Tree and neural network models.

No.	Computation Time	
	Decision Tree Model	Neural Network
1	0.031229	1.040929
2	0.055537	1.110908
3	0.061133	1.175104
4	0.051996	2.634261
5	0.074594	1.068259
6	0.052359	1.24145
7	0.050016	1.313238
8	0.047997	1.392138
9	0.073211	1.600991
10	0.078132	2.092879
11	0.058697	1.389741
12	0.057674	1.334164
13	0.062324	1.848112
14	0.082012	1.639582
15	0.091128	1.899683
16	0.070998	1.397739
17	0.044996	1.257645
18	0.058901	1.204572
19	0.045997	1.127632
20	0.056571	1.180286
21	0.049337	1.2724
22	0.063612	1.046959
23	0.064478	1.25124
24	0.063231	1.501523
25	0.063992	3.656594
26	0.083996	1.15601
27	0.058533	1.216549
28	0.050994	1.071333
29	0.048935	1.203264
30	0.048	1.352984
31	0.047018	1.296979
32	0.081719	1.195881
33	0.045019	1.038161
34	0.052759	1.117537
35	0.048993	1.120578
36	0.063812	1.11737
37	0.053889	1.229534
38	0.054619	1.55222
39	0.050994	1.119644
40	0.060458	1.255233
41	0.051103	1.493202
42	0.050003	1.005049
43	0.05909	1.304165
44	0.052995	1.251586
45	0.061735	1.457659
46	0.050007	1.086125
47	0.052067	1.379029
48	0.051687	1.133587
49	0.050996	1.049925
50	0.044999	1.177412
51	0.064901	1.06846
52	0.056602	0.970769
53	0.051998	1.345009
54	0.046708	1.189245
55	0.047584	1.125525
56	0.089003	1.555476
57	0.0625	2.134213
58	0.086094	2.15842
59	0.068346	1.812286
60	0.059886	1.31289
61	0.0644	1.274483
62	0.053056	1.663544
63	0.077142	1.632083
64	0.084134	2.141111
65	0.074552	1.464199
66	0.057663	1.094026
67	0.048291	1.640016
68	0.044992	1.486279
69	0.049449	1.069333
70	0.046227	1.450437
71	0.063584	1.509127
72	0.075065	1.465219
73	0.047998	1.345517
74	0.047002	1.229406
75	0.056996	1.622626
76	0.048608	1.10312
77	0.046375	1.286845
78	0.047895	1.19502
79	0.054994	1.117199
80	0.074998	1.395097
81	0.05399	1.271263
82	0.053778	1.882056
83	0.065562	1.208384
84	0.055619	1.22781
85	0.047999	1.047544
86	0.048203	1.251664
87	0.04885	1.160577
88	0.050631	0.874186
89	0.055177	1.260275
90	0.049021	1.201123
91	0.044018	1.254685
92	0.046995	1.244649
93	0.047016	1.05943
94	0.052024	0.944234
95	0.042279	1.299954
96	0.059698	1.503714
97	0.047133	1.13383
98	0.060916	1.063454
99	0.047019	0.98657
100	0.05708	1.087645
Average computation time	0.057146	1.348791

**Table 7:** Performance metric of the DT classifier (for the recommended activities).

No	Type of ADHD	Decision Tree					
		TPR	TNR	PR	RC	AC	F1
0	Mix-type (AOCD + AMOD + ACB)	0.98	0	1	0.98	0.99	0.99
1	hyperactivity (AOCD)	1	0	1	1	1	1
2	inattention (AIC)	1	0	1	1	1	1
3	ODD (ACB)	1	0	1	1	1	1
4	Non-ADHD (No)	1	0.02	0.95	1	0.99	0.97
Accuracy average		0.996					

## Data Availability

Data sharing is not applicable.
